# The Evolution of Sodium-Glucose Co-Transporter-2 Inhibitors in Heart Failure

**DOI:** 10.7759/cureus.19379

**Published:** 2021-11-08

**Authors:** Olusayo Fadiran, Chike Nwabuo

**Affiliations:** 1 Internal Medicine, Howard University Hospital, Washington, USA; 2 Internal Medicine, Johns Hopkins Bloomberg School of Public Health, Baltimore, USA

**Keywords:** diabetes type 2, review of clinical trials, heart failure with preserved ejection fraction, heart failure with reduced ejection fraction, sodium-glucose cotransporter-2 (sglt-2) inhibitors

## Abstract

Sodium-glucose co-transporter-2 (SGLT2) inhibitors have evolved over the years, based on data from several randomized, double-blinded, placebo-controlled clinical trials. Formerly used primarily for blood sugar control in patients with diabetes, they are now used to decrease the risk of hospitalization for heart failure (HF), or of death from cardiovascular (CV) causes, in patients with heart failure with reduced ejection fraction (HFrEF). They have also been shown to slow the progression of renal disease and prevent death related to renal causes in patients with chronic kidney disease (CKD). They are currently being studied to decrease the risk of HF hospitalization in patients with preserved ejection fraction subtype and have shown positive results. The transition of SGLT2 from a medication used in diabetes to an established HF medication was a result of the hypothesis generated from the analysis of earlier trials in diabetic patients and further testing of this hypothesis in an HF population. By way of this review, we aim to highlight the rationale for the paradigm shift of SGLT2 inhibitors from their use in diabetic patients to their use in all patients with HF, regardless of the presence of diabetes. To support our recommendation, we'll present detailed results of several major clinical trials and a meta-analysis study that led to this discovery, along with clinical indication for the same.

## Introduction and background

Sodium-glucose co-transporter-2 (SGLT2) inhibitors were initially introduced as an anti-diabetic medication. They have, however, gained widespread use not only as a medication with the ability to lower blood glucose but also to uniquely demonstrate renal protective effects, improve cardiovascular (CV) outcomes, and provide other physical benefits. These array of other potential clinical uses and benefits include weight loss, improved blood pressure, decreased incidence of adverse CV outcomes such as hospitalization for heart failure (HF), decreased CV mortality, decreased incidence of non-fatal myocardial infarction (MI), and stroke in diabetics with known atherosclerotic cardiovascular disease (ASCVD) most notably MI [1], and decreased rate of hospitalization for HF or CV mortality in all patients with heart failure with reduced ejection fraction (HFrEF) [2]. Potential possible renal benefits, particularly in diabetics with chronic kidney disease (CKD) and albuminuria, include a decrease in albuminuria, reduction of the decline of estimated glomerular filtration rate (eGFR) or progression to end-stage renal disease, lower death rates from renal causes, and extended time to initiation of renal replacement therapy [3]. Currently, FDA approved and indicated for HFrEF, the rise to fame of SGLT2 inhibitors did not happen overnight. As optimizing blood sugar control was the initial indication for the medication, earlier trials involved only study participants with diabetes, and findings from their subgroup analysis and outcomes were the rationale for further questions and modification of study participants to investigate specific outcomes of interest to provide further validity. This process led to a shift in indication for their clinical use and also multiple bio-investigations into the possible alternate mechanism of action and effects on cardiac remodeling independent of inhibiting the SGLT2 receptor at the proximal convoluted tubules of the kidneys. The first result, published in 2015, from the EMPA-REG OUTCOME trial, whose primary focus was on the use of empagliflozin in reducing major adverse cardiovascular events (MACE) defined as non-fatal MI, stroke, and CV death in diabetics heralded this evolutionary investigation into SGLT2 inhibitors [4]. Most recently, empagliflozin has been proven to reduce the risk of hospitalization for HF or CV death in all study participants with heart failure with preserved ejection fraction (HFpEF) [5]. Between this time frame, published results from other clinical trials involving other SGLT2 inhibitors, including canagliflozin and dapagliflozin, have demonstrated consistent results in terms of favorable prevention of HF hospitalizations. While other review articles have focused much on summarizing the result of these clinical trials and providing meta-analysis findings in detail, our approach would highlight time stamps of these notable clinical trials while providing a brief summary of the findings that led to further investigation to provide a holistic picture and overview of why SGLT2 inhibitors transitioned from a drug initially used for diabetes to an established HF medication.

## Review

Diabetics with established ASCVD, who were administered 10 mg or 25 mg of empagliflozin over a median treatment duration of 2.6 years in the EMPA-REG OUTCOME trial, had significantly lower rates of MACE described as non-fatal MI, stroke or CV death, hazard ratio (HR) 0.86; 95% CI (0.74-0.99) compared to placebo [4]. The findings from this study represented a new indication for clinical use of SGLT2 inhibitors in diabetics with stable coronary artery disease, history of MI, peripheral artery disease, or stroke. Other studies supporting the result of decreased MACE incidence with SGLT2 inhibitors included results from the CANVAS Program, which showed that diabetic participants with ASCVD or CV risk factors had significantly fewer MACE incidences while on 100 mg or 300 mg of canagliflozin compared to placebo, HR 0.86; 95% CI (0.75-0.97) [6]. MACE reduction was the primary CV benefit and indication for use of SGLT2 inhibitors in diabetics with ASCVD during the time frame of the results from these earlier trials. Interesting results from secondary analysis of other outcomes demonstrated that there were other potential and consistent CV benefits apart from MACE. Empagliflozin reduced the key secondary outcome risk of CV death, HR 0.62; 95% CI (0.49-0.77) or hospitalization for HF, HR 0.65; 95% CI (0.50-0.85) in the EMPA-REG OUTCOME trial [4], which was a similar finding with canagliflozin in the CANVAS Program. Canagliflozin decreased the secondary outcome of CV mortality or hospitalization for HF, HR 0.78; 95% CI (0.67-0.91) and reduced hospitalization for HF, HR 0.67; 95% CI (0.52-0.87) [6]. Although dapagliflozin in DECLARE-TIMI 58 did not significantly reduce the risk of MACE in adults with type 2 diabetes and elevated risk factors or with ASCVD, it was shown to significantly attenuate CV mortality or HF hospitalization, HR 0.83; 95% CI (0.73-0.95) which was a finding most attributable to decreased HF hospitalization, HR 0.73; 95% CI (0.61-0.88) [7]. The CREDENCE trial was also shown to reveal a consistent key secondary outcome in participants with type 2 diabetes and diabetic nephropathy of decreased HF hospitalization or CV death, HR 0.69; 95% CI (0.57-0.83), decreased HF hospitalization, HR 0.61; 95% CI (0.47-0.80), and decreased MACE, HR 0.80; 95% CI (0.67-0.95) [3]. Arnott C et al. performed a meta-analysis of the four major trials, EMPA-REG OUTCOME, CANVAS Program, CREDENCE, and DECLARE-TIMI 58. Of the efficacy outcome studied including MACE, CV death, fatal and non-fatal MI, fatal and non-fatal stroke, HF hospitalization, CV death or HF hospitalization, and all-cause mortality, SGLT2 inhibitors had the greatest impact on reducing hospitalization for HF with an overall 32% reduction compared with placebo, HR 0.68; 95% CI (0.60-0.76) without evidence of heterogeneity between these studies [8]. These earlier trials demonstrating reduced HF hospitalization or CV death were performed exclusively in type 2 diabetes participants, however, subgroup analysis of diabetics with the previous diagnosis of HF suggested that this finding might benefit patients more with a history of HF most notably HFrEF. This approach was compromised by the fact that the subgroup of participants with HF represented a minority of the study size with diabetes and was not investigated accurately enough because they were not the primary objective of the design [9,10]. For this reason, there was a need to define a specific study population of patients with HF by including study selection criteria such as ejection fraction and N-terminal-pro hormone B-type natriuretic peptide levels.

**Figure 1:**
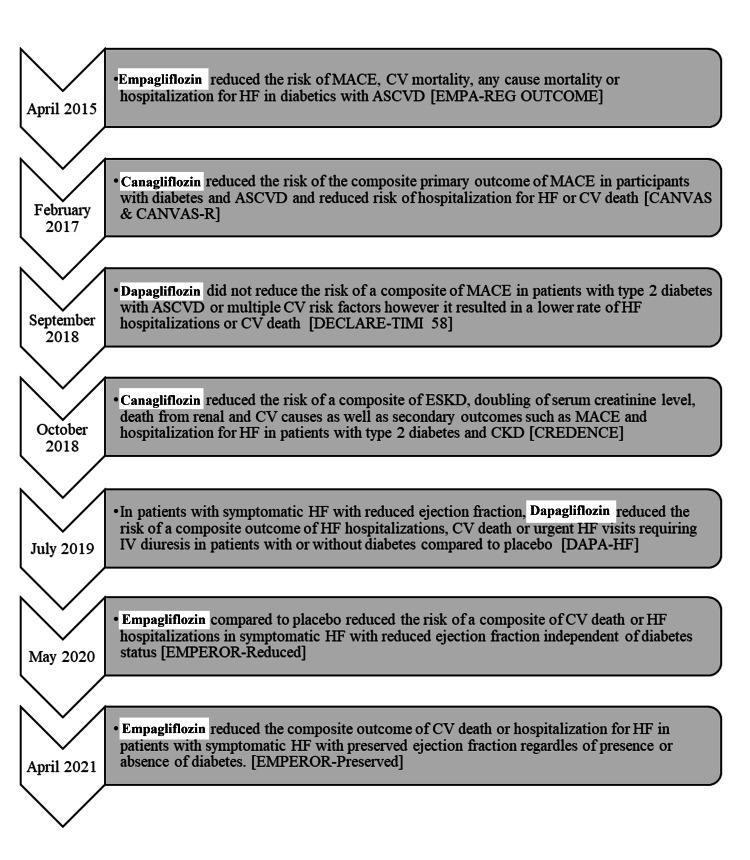
Timeline and summary of major landmark trials involving SGLT2 inhibitors. Month and year represent the actual study completion period in which the last participant in the clinical study was examined to collect final data for the primary outcome, secondary outcome measures, and adverse effects. Trial name in []; SGLT2-inhibitor type highlighted in bold. MACE: Major adverse cardiovascular events which are a composite outcome of nonfatal myocardial infarction, stroke, and CV mortality; CV: Cardiovascular; ASCVD: Atherosclerotic Cardiovascular Disease; HF: Heart Failure; CKD: Chronic kidney disease; ESKD: End-stage kidney disease.

SGLT2 inhibitors were hypothesized to benefit patients with HF, particularly HFrEF defined as an ejection fraction less than 40%. Dapagliflozin and Prevention of Adverse Outcomes in Heart Failure (DAPA-HF) was the first study involving the use of SGLT2 inhibitors in symptomatic patients with HFrEF to evaluate the effect of dapagliflozin on the outcome of worsening HF, hospitalization for HF, or CV death [11]. All participants had established HFrEF with a mean EF of about 30% and were previously on guideline-directed HF medications consisting of angiotensin-converting enzyme inhibitors (ACEIs), angiotensin-receptor blockers (ARBs), or sacubitril-valsartan with a beta-blocker (BB) and a mineralocorticoid receptor antagonist (MRA) if tolerated. The results of the DAPA-HF trial were followed subsequently by the EMPEROR-Reduced trial: a similarly constructed clinical trial involving a relatively greater severity of decreased left ventricular (LV) systolic function, mean EF of less than 30% [12]. In both trials, SGLT2 inhibitors reduced the composite outcome of HF hospitalization or CV death, DAPA-HF: HR, 0.74; 95% CI (0.65 to 0.85) and EMPEROR-Reduced: HR, 0.75; 95% CI (0.65 to 0.86) although the duration of follow-up was relatively shorter in the EMPEROR-Reduced trial. Hypoglycemic adverse events defined as plasma glucose level less than 70 mg/dl or symptoms that required intervention were more common in diabetic participants on empagliflozin (2.2%) than those without diabetes (0.7%) in the EMPEROR-Reduced trial [12]. This infers that chronic HFrEF patients without diabetes are unlikely to develop hypoglycemia compared to diabetics with HFrEF particularly when the latter concomitantly administer insulin. The most consistently reported adverse effects among study participants have been hypotension, dehydration, genital yeast infection, and UTI [11,12]. On May 5, 2020, dapagliflozin was approved by the US FDA to reduce the risk of HF hospitalization and CV deaths in adults with chronic New York Heart Association (NYHA) class II-IV HFrEF. This approval was followed subsequently by a similar FDA-approved indication for the use of empagliflozin. Figure 1 shows time stamps of trial completion and a summary of findings of major SGLT2 inhibitor landmark trials.

In the EMPEROR-Preserved trial, empagliflozin, at a dose of 10 mg daily, decreased the composite primary outcome of HF hospitalization or CV death in HFpEF, HR 0.79; 95% CI (0.69-0.90) which was mainly attributable to statistically significant reductions of hospitalization for HF, HR 0.71; 95% CI (0.60-0.83) [5]. This finding was consistent in both participants with or without diabetes. Although their efficacy was diminished by stratifying according to increasing ejection fraction subgroups. This is an important study result in HFpEF because previously known popular HF medications such as the BBs, ACEIs, and ARBs, have not been shown to improve outcomes in this subtype of HF. The proposed mechanisms of action of SGLT2 inhibitors in HF patients include but are not limited to diuresis from glycosuria and natriuresis as a result of inhibition of the sodium-glucose transporter in the proximal renal tubule, improving the energy source for cardiac metabolism of the failing heart by aiding the liver in ketogenesis, possible anti-inflammatory properties and hindering adverse cardiac remodeling [13]. Although, not the focus of our review, of great importance is the extensive renal protective benefits that were shown in the listed trials above. SGLT2 inhibitors were shown to decrease albuminuria and the progression of CKD in diabetics with nephropathy, in addition to decreasing the renal composite outcome of end-stage kidney disease (ESKD), doubling of serum creatinine, and renal or CV death in the CREDENCE trial. SGLT2 inhibitors also decreased the key secondary renal composite outcome in the EMPEROR-Reduced trial. The progression of CKD defined by the change in slope of mean eGFR was diminished by empagliflozin in participants with CKD in both the EMPEROR-Reduced and EMPEROR-Preserved trial. Table 1 gives a brief description of the characteristics of the major SGLT2 inhibitor trials.

**Table 1:** Major clinical trials involving SGLT-2 inhibitors. ASCVD: Atherosclerotic cardiovascular disease; MI: Myocardial infarction; CV: Cardiovascular; HF: Heart Failure; ESKD: End-stage kidney disease; CKD: Chronic kidney disease; NYHA: New York Heart Association; HFrEF: Heart failure with reduced ejection fraction; HFpEF: Heart failure with preserved ejection fraction; KCCQ: Kansas City Cardiomyopathy Questionnaire; eGFR: Estimated glomerular filtration rate; 3-point MACE: Non-fatal MI, non-fatal stroke or CV death; 4-point MACE: Non-fatal MI, non-fatal stroke, CV death, and unstable angina hospitalization; SGLT-2: Sodium-glucose co-transporter-2. Renal composite outcome: ≥40% decrease in eGFR to <60ml/min/1.73m2, ESKD, or death from renal or cardiovascular causes.

Clinical trial	Study design	SGLT-2 inhibitor type	Study participants	Primary outcome	Secondary outcome	Median study follow-up (Y)
EMPA-REG OUTCOME	Randomized, parallel, multicenter double-blinded, placebo-controlled, phase III	Empagliflozin 10 mg or 25 mg	7028 adults with type 2 diabetes and ASCVD	3-point MACE	4-point MACE, HF hospitalization, Silent MI, any cause death, CV death, and nonfatal stroke	3.1
CANVAS and CANVAS-R	Randomized, parallel, multicenter, double-blinded, placebo-controlled, phase IV	Canagliflozin 100 mg or 300 mg	10142 adults with type 2 diabetes with ASCVD or risk factors	3-point MACE	Any cause death, CV death, progression of albuminuria, composite CV death or HF hospitalization	2.4
DECLARE-TIMI 58	Randomized, parallel, multicenter, double-blinded, placebo-controlled, phase III	Dapagliflozin 10 mg	17160 adults with type 2 diabetes with ASCVD or risk factors	3-point MACE, composite CV death, or HF hospitalization	Renal composite outcome, any cause death, HF hospitalization, and CV death	4.2
CREDENCE	Randomized, parallel, multicenter, double-blinded, placebo-controlled, phase III	Canagliflozin 100 mg	4401 adults with type 2 diabetes, stage 2 or 3 CKD and macroalbuminuria	Composite ESKD, doubling serum creatinine, renal or CV death	CV death or HF hospitalization, 3-point MACE, HF hospitalization, CV death, and any cause death	2.6
DAPA-HF	Randomized parallel, multicenter, double-blinded, placebo-controlled, phase III	Dapagliflozin 10 mg	4744 adults with NYHA class II-IV HFrEF	Composite HF hospitalization/urgent visit or CV death	CV death or HF hospitalization, Change in KCCQ symptom score, and any cause death	1.5
EMPEROR-Reduced	Randomized, parallel, multicenter, double-blinded, placebo-controlled, phase III	Empagliflozin 10 mg	3730 adults with NYHA class II-IV HFrEF	Composite CV death or HF hospitalization	Total HF hospitalization, mean slope of change in eGFR, any cause death, change in KCCQ symptom score, new-onset diabetes	1.3
EMPEROR-Preserved	Randomized, parallel, multicenter, double-blinded, placebo-controlled, phase III	Empagliflozin 10 mg	5988 adults with NYHA class II-IV HFpEF	Composite CV death or HF hospitalization	Total HF hospitalizations, mean slope change in eGFR, change in KCCQ symptom score, and any cause death	2.2

While the above-listed studies represent a fraction of completed clinical trials involving SGLT2 inhibitors, they reflect the largest powered sample-sized studies with clinically important results. Other similar trials, such as DEFINE-HF [14], EMPERIAL-Preserved, and EMPERIAL-Reduced [15] had smaller study enrollment ranging from a sample size of 263 to 312 participants. The VERTIS CV trial, which was highly powered to investigate ertugliflozin in 8238 patients with type 2 diabetes and ASCVD yielded non-inferior outcomes of MACE and CV death or HF hospitalizations [16]. However, positive outcomes of decreased HF hospitalizations or CV death were shown in the SOLOIST-WHF [17] and the recently concluded PRESERVED-HF trial. Future trials such as the CHIEF-HF and the DELIVER trial are likely to provide more information that would continue to reshape and solidify this novel emerging role. We uniquely provided details in a historical, time progressive fashion with the hope of providing a rationale for why one clinical trial led to another and how results of study findings modify clinical indications for drug use. Digging into the details of each individual clinical trial, such as study design, safety outcomes and adverse effects of each SGLT2 inhibitor was beyond the scope of our review and may represent a limitation of this study. We, however, met our objective by simplifying and providing an overview of the thought process into this current trend and novel indication for SGLT2 inhibitors.

## Conclusions

Initially indicated to be used for the management of diabetes, SGLT2 inhibitors have proven to be effective across the board for all HF patients. This review discussed how it all started and why this change happened. We also provided a snapshot of the major practice-changing clinical trials. In the current trend of investigations into SGLT2 inhibitors, the main outcome of interest continues to remain its ability to decrease the rate of hospitalization for HF patients and improve CV mortality. However, there is a potential for a wider array of other hidden effects and clinical benefits of this medication. These potential outcomes include improving the health status, quality of life, and functional status of patients with symptomatic HF and inducing structural changes in the failing heart that would forestall or reverse adverse cardiac remodeling. However, this can only be discovered by continuously embarking on goal-directed investigations. What we know at this moment about the endocrine, CV, and renal benefits might just be the tip of the iceberg. SGLT2 inhibitors have shown promise of being an extremely versatile medication with a brighter tomorrow.
